# Towards sustainable management of cassava mosaic disease: The impact of awareness campaigns in Benin

**DOI:** 10.1016/j.jafr.2025.101827

**Published:** 2025-06

**Authors:** Dèwanou Kant David Ahoya, Jacob Afouda Yabi, Jerome Anani Houngue, Serge Sètondji Houédjissin, Martine Zandjanakou-Tachin, Ettien Antoine Adjei, Eveline Marie Fulbert Windinmi Sawadogo-Compaore, Justin Simon Pita, Corneille Ahanhanzo

**Affiliations:** a Laboratory for Analysis and Research on Economic and Social Dynamics (LARDES), University of Parakou (UP), Benin; b Central Laboratory of Plant Biotechnology and Plant Improvement (LCBVAP), University of Abomey-Calavi (UAC), Benin; c Research Unit in Horticultural Production and Green Space Management (URPHGEV) of the School of Horticulture and Green Space Management (EHAEV), National University of Agriculture (UNA), Benin; d Central and West African Virus Epidemiology (WAVE), Scientific and Innovation Hub of Bingerville, Félix Houphouët-Boigny University (UFHB), Bingerville, Côte d'Ivoire; e Department of Natural Resource Management and Production Systems, Institute of Environment and Agricultural Research (INERA), Burkina Faso

**Keywords:** Awareness campaign, Cassava mosaic disease, Impact assessment, Propensity score matching (PSM), Benin

## Abstract

Transboundary diseases, such as cassava mosaic disease (CMD), represent a significant risk to food security and the livelihoods of millions of households in sub-Saharan Africa. To address this issue, awareness campaigns have been conducted targeting farmers and stakeholders within the cassava secor. The objective of this study is to quantify the impact of these awareness campaigns on the knowledge of CMD, the adoption of management practices, and the incidence of the disease in cassava fields. A random sample of 305 farmers and 77 cassava fields in Benin was selected for data collection. To account for potential selection bias associated with observable characteristics, we applied Propensity Score Matching (PSM). The results indicate that farmers who participated in the training demonstrated significantly higher levels of CMD knowledge and were more likely to adopt a greater number of management practices, which ultimately led to a reduction in the prevalence of the disease in their fields. However, CMD symptoms were still prevalent in the majority (61,04) of cassava farms, regardless of participation in the campaigns, due to the lack of healthy planting material and the abundance of whiteflies. These findings suggest that awareness campaigns can significantly improve farmers' knowledge and encourage behavioural changes in the identification and adoption of sustainable CMD management practices. It also shows the need to provide famers with healthy cuttings for more effective disease management.

## Highlights

• Cassava production is threatened by Cassava Mosaic Disease (CMD).
• Awareness campaigns have increased farmers' knowledge and adoption of CMD management practices.
• CMD management practices help reduce disease incidence in cassava fields.
• Awareness campaigns are key to effective CMD management.
• Stakeholders must prioritize promoting awareness campaigns for effective CMD management.

## Graphical abstract

**Figure:**
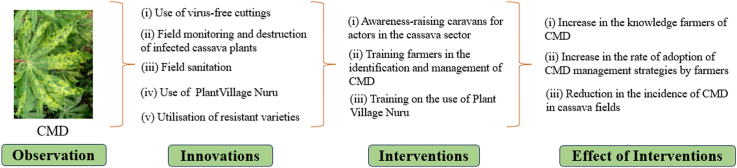

## 1 Introduction

Cassava (*Manihot esculenta*, Crantz) is a perennial woody shrub belonging to the *Euphorbiaceae* family [1]. Native to South America, it is now widely cultivated in Benin, spanning the entire national territory and representing the second most extensive crop in terms of cultivated area, following maize [2]. Cassava plays an important role in human nutrition as a food source (attiéké, tapioca, biscuits, lafun, and gari), and in industry, where it serves as a key raw material for the production of starch, glucose, biofuels, and ethanol [3]. Additionally, it is a staple feed for livestock [4].

Despite its significant potential to generate income for small-scale farmers and its contribution to the development of sub-Saharan Africa [5,6], cassava production faces numerous challenges. Among these, viral diseases represent a major constraint, with Cassava Mosaic Disease (CMD) being one of the most pervasive threats [7]. CMD is caused by a group of viruses known as begomoviruses, belonging to the *Begomovirus* genus within the *Geminiviridae* family [8]. These viruses are transmitted by the whitefly, *Bemisia tabaci*, which is the main vector, as well as through the propagation of infected cassava cuttings by human [9,10]. Notably, the Cassava Mosaic Virus (CMV) transmission occurs predominantly via vegetative means, with infected stem cuttings being the major source of disease spread, rather than transmission via whiteflies [11].

The management of CMD is particularly challenging due to the systemic nature of the infection, the lack of effective chemical control measures, the dependence on specific vectors, and the predominance of preventive rather than curative control strategies. In practice, farmers often source planting materials from their own farms or neighbouring fields [11,12], facilitating the spread of the disease. Furthermore, it has been documented that farmers occasionally transport infected cassava cuttings over long distances, contributing to the emergence of new CMD hotspots or epicenters [12]. This situation compromises the effectiveness of control measures deployed to combat this viral disease [[13], [14], [15]].

Moreover, a lack of knowledge among farmers regarding the identification of CMD symptoms, its transmission mode, management pactices, and its impact on cassava yield has been observed [11,16]. This knowledge gap leads to the non-application of management practices, resulting in a high incidence of CMD in cassava farms [17]. Thus, it is crucial to improve farmers' understanding of CMD, which can be achieved through the implementation of training programmes, awareness campaigns and capacity-building by extension agents and research institutions [11,12,18].

In this context, the Central and West African Virus Epidemiology (WAVE) programme has been conducting awareness-raising and training activities for farmers since its inception in 2015. These initiatives aim to improve farmers’ knowledge of the disease (symptoms, etiology, causes, consequences) and promote the adoption of effective management practices [19]. Six (6) CMD management practices have been widely disseminated among the beneficiary communities, including the (i) use of healthy cuttings, (ii) regular monitoring of cassava fields and uprooting/destruction of infected plants, (iii) regular fields sanitation, (iv) respect of planting density, (v) use of resistant varieties, and (vi) real-time phytosanitary diagnostics of plants showing disease symptoms through the PlantVillage Nuru intelligent application [[20], [21], [22]].

However, existing literature indicates that similar approaches have already been used in the management of diseases concerning other crops, such as avocado [23], maize [24], vegetables [25], mango [26], and banana [27]. In these cases, farmers have been sensitized via various communication channels, such as Farmer Field Schools [25], plant clinics [28] and, in some cases, ICTs have been used to disseminate disease-related information [29]. These awareness initiatives have been evaluated using econometric models, such as multinomial logistic regression [23], difference-in-difference method [25,26], and propensity score matching [24,29]. Other methods, such as household fixed effects and descriptive statistics, have also been employed [27]. While applied in different contexts, these methods rely on comparing treated and control populations [[23], [24], [25], [26],^29^]. Overall, the results of these studies indicate either a positive effect or a combined effect of the innovations disseminated through awareness activities, both on the knowledge, attitudes and disease management practices, as well as on the yield and income of farmers [[23], [24], [25], [26], [27], [28]].

Despite these findings, such studies rarely account for disease incidence dynamics when evaluating impact. Also, in the case of WAVE's awareness campaigns on CDM in Benin, no impact evaluation has been conducted. Furthermore, the only available data on CMD knowledge and management practices in the Benin context come from Houngue et al. [17], which are limited to a basic description of the practices adopted by farmers to combat this disease. Given this, and considering the threat posed by CMD, as well as the socio-economic importance of cassava cultivation for rural households, it is essential to analyze the impact of the capacity-building activities carried out by WAVE. This will provide policymakers with reliable, robust data for more effective and informed disease control in Benin. This study addresses this need, aiming to evaluate the impact of WAVE's awareness campaigns on CMD management by farmers using propensity score matching (PSM). Specifically, we examine the relationship between participation in awareness campaigns and various outcome variables related to disease management. Our approach takes into account the knowledge of CMD, the adoption of recommended management practices and, unlike previous studies, integrates disease incidence in cassava fields into the analysis.

The following section outlines the structure of the remainder of the article. The subsequent section describes the intervention under study, the data collected, and the statistical matching method applied to quantify the impact. The results of the estimations are presented and discussed in sections three and four. Finally, section five concludes by addressing some policy implications.

## 2 Data and methods

### 2.1 Studied intervention

The intervention examined in this study is an awareness campaign aimed at enhancing the understanding of farmers of the risks associated with cassava viral diseases. This initiative was implemented by the Central and West African Virus Epidemiology (WAVE) programme in 2021, between March and August. During these awareness campaigns, WAVE researchers disseminated critical information to stakeholders within the cassava sector, focusing on various aspects of CMD, including its symptoms, causes, modes of transmission, impact on yield and food security, as well as prevention and control practices. The principal prevention and control measures communicated during these campaigns were as follows: (i) the use of healthy and virus-free cuttings for planting, (ii) regular field surveillance and the removal of infected cassava plants, (iii) respect of planting density, and (iv) the use of the PlantVillage Nuru mobile application for the real-time detection of CMD.

**Fig. 1:**
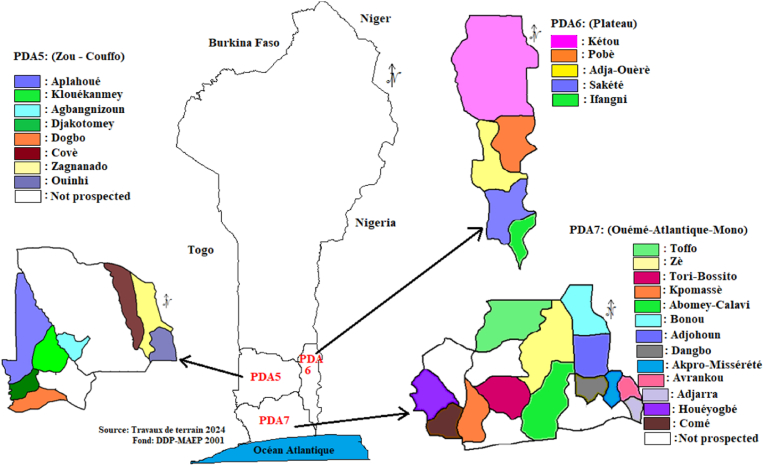
Study area map.

To reach a broad audience, WAVE researchers in Benin primarily employed the use of training and awareness caravans. These events were organised in Agricultural Development Poles (PDA) 5, 6, and 7, to raise awareness and provide training for stakeholders in the cassava sector. These three PDAs were selected due to their suitability for cassava production, accounting for over 60 % of the national cassava production [30]. Fig. 1 presents the map of the study area. The participants included farmers, cutting multipliers, agricultural advisors, and policymakers, all of whom are stakeholders concerned with the risks posed by CMD. During these sessions, PowerPoint presentations were delivered to address different aspects of CMD, including its geographical distribution, propagation mechanisms, symptoms, surveillance, and management strategies. Additionally, field visits were conducted to demonstrate the use of the PlantVillage Nuru application for real-time disease detection in cassava crops.

The estimated audience for this awareness campaign organised by WAVE in Benin, was more than five thousand (5,000) farmers. These efforts aim to bolster the resilience of the cassava sector and contribute to enhanced food security in rural communities.

### 2.2 Data and sample

The data for this study were collected from 26 municipalities in 6 departments, encompassing Agricultural Development Poles 5, 6, and 7 in Benin (Fig. 1). For each selected municipalities, a list of cassava farmers who participated in the awareness campaigns was obtained from the Central Laboratory of Plant Biotechnology and Crop Improvement (LCBVAP) at the University of Abomey-Calavi, which serves as the WAVE hub in Benin. For farmers who did not participate in the awareness campaigns, the list was obtained from the Municipality Cell Heads (CCeC). Based on a purposive sampling approach, farmers were subsequently selected randomly, considering their availability. The sample size in each municipalities was determined based on the number of farmers who responded to the calls made by the Municipality Cell Heads of the respective municipalities (Appendix Table A1). A total of 305 cassava farmers was surveyed, comprising 85 farmers who had participated in the awareness campaigns conducted by WAVE (treatment group or participants) and 220 farmers who had not participated in these campaigns (control group or non-participants). To ensure comparability between the two groups in terms of socio-economic characteristics, participants and non-participants were selected from the same municipalities. The ratio of participants to non-participants was approximately 1 to2.59, with a higher number of respondents in the control group to create an adequate matching [31]. Following the administration of the questionnaire to the farmers, a random survey was conducted on selected cassava fields to assess the incidence of CMD. In total, 77 fields were visited, comprising 31 belonging to participants and 46 to non-participants.

The survey of cassava farmers was conducted by enumerators using tablets equipped with the KoboCollect application to administer the questionnaire. Prior to the data collection, the selected enumerators underwent comprehensive training on data collection methods and conducted a pilot test by interviewing farmers not included in the study. This preliminary phase allowed for the evaluation of the relevance of the questionnaire and ensured that the collected data were appropriate to achieve the study's objectives. During the actual data collection, the objectives and importance of the survey were clearly explained to the cassava farmers before administering the questionnaire, in order to obtain their informed consent for participation. To ensure the accuracy of the data collected, and in accordance with the methodology of Kombo et al. [32], local interpreters were mobilized to facilitate communication with the farmers, taking into account the ethnic diversity in the study area.

The questionnaire collected information on a range of topics, including household characteristics, participation in awareness campaigns, access to institutional services, cassava production systems, cutting exchange methods, and farmers' knowledge and management of CMD. Additionally, to assess the phytosanitary status of the fields and in line with the harmonised WAVE protocol [17], we conducted a visual assessment of CMD symptom expression on the leaves, using the severity scale defined by Hahn et al. [33] to estimate the incidence of CMD.

### 2.3 Outcome variables

The data for this study were collected in February 2024, prior to the commencement of the 2024–2025 agricultural season. The outcome variables employed for the evaluation of the impact of the awareness campaigns are as follows:

*General knowledge of CMD (score)*: The level of farmers' knowledge about CMD was assessed using five questions. The first question evaluated the farmers' capacity to identify the symptoms of the disease. They were presented with two images of cassava plants and were required to ascertain the health status of each plant, indicating whether it was diseased or healthy. The objective of this initial question was to ascertain the farmers' ability to recognize the symptoms of CMD. The remaining four knowledge questions were focused on the disease name displayed in the diseased photo, its cause, modes of transmission, and CMD management strategies. For each question, a value of one (1) was assigned if the farmer provided the correct answer, and zero (0) otherwise. The aggregate score for the five knowledge test questions was designated as the general knowledge of CMD variable. This score represents the primary variable of interest in terms of CMD knowledge. In addition to the aggregate score, the estimation results for the five individual knowledge questions were also presented, as they cover different aspects of the information related to the CMD awareness campaign.

*Adoption of CMD management practices (score)*: One of the principal messages conveyed during the awareness campaigns was the necessity of employing a combination of practices to combat CMD. Consequently, the number of recommended CMD management practices adopted by a farmer was used as one of the main outcome variables [34]. For each recommended practice, a value of one (1) was assigned if the farmer used it, and zero (0) otherwise. The total score for the adoption of CMD management strategies for each farmer was obtained by summing the values assigned to each management practice.

*Incidence of CMD:* The incidence of CMD is defined as the ratio of infected plants or fields to the total number of plants or fields evaluated. It is typically expressed as a proportion (ranging from 0 to 1) or as a percentage (ranging from 0 to 100 %) of infected plants or fields [35]. This allows for the quantification of the prevalence of the disease within a given population. In the present study, the incidence of CMD was estimated through a method of counting the number of infected plants and evaluating the number of fields visited. The severity of CMD was assessed on a scale of 1–5, according to the severity scale described by Ref. [33]. Level 1 corresponds to no infection, level 2 to mild infection, level 3 to moderate infection, level 4 to severe infection, and level 5 to very severe infection.

It is important to note that, due to the limited number of fields surveyed per group, the incidence of CMD was excluded from the PSM analysis regarding the differential effects of participation in the awareness campaigns. This exclusion was necessary to avoid issues related to insufficient statistical power and low overlap between campaign participants and the comparison group.

### 2.4 Empirical approach

In the context of impact evaluations, one of the principal challenges is determining what would have occurred in the absence of awareness campaigns conducted by WAVE. The observed outcome among beneficiaries in the absence of the intervention is referred to as the counterfactual. In order to attribute any differences in the outcomes of interest to the awareness campaign, the control group (non-participants) must be comparable to the treatment group (campaign participants), except for the fact that they did not receive the awareness campaign messages. Thus, the key issue lies in identifying comparable groups.

In addressing this issue, the experimental method is often considered the gold standard [36,37]. It allows for the creation of comparable groups by assuming that, prior to the commencement of the awareness campaign, farmers are randomly assigned to a treatment group (comprising those who will participate in the CMD campaign through one of the communication channels) and a control group (comprising those who will not participate in the campaign). This approach generates a robust counterfactual. Nevertheless, the experimental approach was not applicable in this instance, given that participation in the awareness campaigns was not randomly assigned. Consequently, farmers could decide to participate or decline based on a range of observable and unobservable characteristics. In other words, participants in the awareness campaigns may exhibit systematic differences from non-participants, and a simple comparison of outcome variable means between the two groups could lead to bias [38]. To address this potential selection bias when estimating the impact of awareness campaigns, the propensity score matching (PSM) method was employed [39]. This quasi-experimental method relies on a statistical model to identify the control or counterfactual group based on observable characteristics. In essence, it involves selecting from a large pool of individuals those who are similar to the treated farmers in terms of observable characteristics that are unaffected by the campaign. Each participant is then matched to a non-participant with similar characteristics using the propensity score.

The implementation of Propensity Score Matching (PSM) is comprised of three distinct steps, as outlined by Ref. [40]. In the initial stage of the process, a probit regression was utilised to generate the requisite propensity scores. These scores are used to estimate the probability of a farmer participating in awareness campaigns. The covariates included in the probit regression are significant pre-treatment variables that may influence participation in the campaigns, as well as outcome variables. In this study, we draw inspiration from the existing literature on the determinants and impacts of participation in plant clinics and other extension programmes [24,28,41,42]. These covariates encompass household demographic characteristics (age, gender, marital status, primary occupation, experience in cassava production, and farmer's education level), farmer assets (area dedicated to cassava cultivation), and access to institutional services (membership in a cassava producers' association and access to extension services). A detailed description of these conditioning variables is presented in Table 1.

**Table 1:** Socio-economic characteristics of interviewed farmers.

Variables	Total sample (n = 305)	Participants (n = 85)	Non-Participants (n = 220)	*t*-test
	Mean (Standard deviation)			
Age	44.92 (11.85)	47.26 (11.58)	44.02 (11.85)	0.032∗∗
Gender (1 = Male)	0.82 (0.38)	0.86 (0.35)	0.82 (0.38)	0.399
Level of education	6.51 (5.47)	7.41 (4.83)	6.16 (5.66)	0.072∗
Marital status	1.98 (0.21)	1.99 (0.11)	1.97 (0.23)	0.555
Primary occupation	0.89 (0.31)	0.91 (0.29)	0.89 (0.31)	0.703
Area dedicated to cassava cultivation	2.63 (3.95)	2.89 (3.48)	2.52 (4.13)	0.465
Agricultural extension	0.64 (0.48)	0.74 (0.44)	0.61 (0.48)	0.036∗∗
Member of a Cassava farmers' Association	0.61 (0.49)	0.68 (0.47)	0.58 (0.50)	0.092∗
Experience in cassava production.	10.24 (2.73)	10.75 (2.28)	10.02 (2.86)	0.036∗∗
Household size	8.26 (4.86)	8.86 (5.59)	8.02 (4.52)	0.177

∗∗∗p < 0.01, ∗∗p < 0.05, ∗p < 0.1.

The second step is to define the common support region, which is the area where the propensity scores of the treatment and control groups overlap. This region extends from the minimum propensity score of farmers who participated in the campaigns to the maximum propensity score of farmers who did not participate. The final step involves matching campaign participants with non-participants using the propensity scores obtained in the first step. The matching algorithm employed is Nearest Neighbour Matching (NNM). To assess the robustness of the results, two alternative matching algorithms were also employed: radius matching with a caliper of 0.05 and kernel matching with a default bandwidth of 0.06. Following the completion of matching quality tests, the impact estimation is performed using the Average Treatment Effect on the Treated (ATT).

$$ATT=E\left\{Y\left(1\right)-Y\left(0\right)|P_{cs}=1\right\}=E\left\{Y\left(1\right)|P_{cs}=1\right\}-E\left\{Y\left(0\right)|P_{cs}=1\right\}$$

where Y(1) et Y(0) represent the outcomes for participants and non-participants in the awareness campaigns, respectively; $P_{cs}$ represents participation in the awareness campaigns, while E{ } represents the mathematical expectation operator.

The ATT is defined as the mean difference in outcomes between campaign participants and non-participants, matched within the common support region. This method is contingent upon the assumption of conditional independence or no confounding, which posits that all variables that are simultaneously influencing the participation decision and the outcome variables must be taken into account. However, this assumption cannot be tested because the data do not provide information on the distribution of untreated outcomes and vice versa [43]. The estimation of treatment effects through matching estimators is based on the assumption of observable selection. Consequently, the potential for unobserved bias to emerge if unobserved variables exert influence upon both treatment assignment and the outcome must be considered. In order to assess the sensitivity of our ATT estimates to a potential violation of the conditional independence assumption, we employed Rosenbaum's bounds test [44].

It is important to highlight that a range of quasi-experimental methods are used to assess the impact of interventions. These include Ordinary Least Squares (OLS), the Heckman two-step selection method, Instrumental Variable (IV) approaches, and Propensity Score Matching (PSM) methods. According to Ref. [45], OLS and IV methods impose a linear form, and the search for valid instruments can be complex in impact analysis. Unlike observed control variables, instrumental variables do not have a direct effect on the outcome. Taking all these factors into account, the decision was made in favour of PSM. This approach helps mitigate biases and provides a more precise evaluation.

## 3 Results

### 3.1 Descriptive statistics

Table 1 presents the descriptive statistics of the socio-economic characteristics of the interviewed farmers, categorised based on their participation or non-participation in WAVE's awareness campaigns. The sample is predominantly comprised of married male farmers residing in households with an average of 8 individuals. The typical farmer is 49 years old and has received only six years of formal education. Cassava farms in the study area are relatively small, with an average size of approximately 2.63 ha. Furthermore, the study revealed that the majority of farmers engage in cassava production as their primary activity and have over 10 years of experience cultivating this crop. Additionally, most farmers (64 %) have access to extension services.

Table 2 presents the descriptive statistics on knowledge and adoption of CMD management practices, distinguishing between participants and non-participants. Regarding CMD knowledge, the majority of farmers (97 %) correctly identified the disease symptoms. However, only 31 % of farmers were able to correctly name the disease, either in French or the local language. Furthermore, farmers demonstrated limited understanding of the cause and modes of CMD transmission. On average, campaign participants exhibited greater knowledge about the disease name (41 %), its cause (31 %), and modes of transmission (38 %) compared to non-participants. The knowledge gain related to CMD was significantly higher among campaign participants.

**Table 2:** Descriptive statistics of CMD knowledge and adoption of management practices.

Outcome variables	Total sample (n = 305)	Participants (n = 85)	Non-participants (n = 220)	*t*-test
	Mean (Standard deviation)			
**CMD knowledge**				
Identification of CMD symptoms (from photo)	0.97 (0.14)	0.98 (0.13)	0.96 (0.15)	0.420
Knowledge of the name of CMD	0.31 (0.46)	0.41 (0.50)	0.27 (0.45)	0.020∗∗
Knowledge of the causes of CMD	0.21 (0.41)	0.31 (0.46)	0.17 (0.038)	0.010∗∗
Knowledge of the modes of propagation of CMD	0.28 (0.37)	0.38 (0.32)	0.24 (0.38)	0.002∗∗∗
Knowledge of CMD management practices	0.42(0.38)	0.60 (0.27)	0.35 (0.32)	0.000∗∗∗
General knowledge related to CMD	0.44 (0.23)	0.53 (0.16)	0.40 (0.25)	0.000∗∗∗
**Adoption of CMD management practices**				
Adoption of healthy cuttings	0.29 (0.45)	0 0.4 (0.49)	0.24 (0.43)	0.005∗∗∗
Adoption of regular field monitoring	0.51 (0.50)	0.85 (0.36)	0.39 (0.49)	0.000∗∗∗
Adoption of field weeding	1 (0)	1 (0)	1 (0)	–
Adoption of recommended planting density	0.51 (0.50)	0.52 (0.50)	0.51 (0.50)	0.950
Adoption of the PlantVillage Nuru application	0.14 (0.35)	0.41 (0.50)	0.04 (0.19)	0.000∗∗∗
General adoption of CMD management practices	0.49 (0.21)	0.64 (0.19)	0.43 (0.19)	0.000∗∗∗

∗∗∗p < 0.01, ∗∗p < 0.05, ∗p < 0.1.

In terms of the adoption of CMD management practices, Table 2 reveals that certain practices are observed with notable infrequency. For example, the utilisation of healthy cuttings (29 %) and the PlantVillage Nuru application (14 %) is relatively limited. However, the majority of farmers (51 %) monitor their cassava fields and respect recommended planting densities (51 %). All surveyed farmers perform at least three field cleanings before cassava harvest. Participants in the campaign have monitored their cassava fields with greater diligence to detect the presence of CMD and have employed a greater number of disease management practices, including the use of healthy cuttings and the PlantVillage Nuru application, compared to their non-participating counterparts. Finally, participants have adopted a significantly higher number of practices on average (3.17) than non-participants (2.17).

### 3.2 Incidence of CMD

The results revealed that CMD symptoms were observed in the majority (61.04 %) of surveyed fields, both among participants (58.06 %) and non-participants (63.04 %) (Table 3). However, this difference was not statistically significant (T-test = 0.67).

**Table 3:** Fields affected by CMD by type of interviewed farmers.

Cassava fields	Number of surveyed fields	Number of infected fields	Incidence (%)
Participants	31	18	58.06
Non-Participants	46	29	63.04
Total	77	47	61.04
T-test	0.67		

Furthermore, the incidence of CMD varied depending on participation in awareness campaigns, ranging from 10.54 % to 10.65 % (Table 4). In the study area, these incidence levels are moderate and fall between 0 % and 25 %.

**Table 4:** CMD incidence by type of interviewed farmers.

Cassava fields	Number of surveyed fields	Number of evaluated plants	Number of plants infected by CMD	Incidence of CMD (%)
Participants	31	930	98	10.54
Non-Participants	46	1380	147	10.65
Total	77	2310	245	10.61

### 3.3 Effect of awareness campaigns on knowledge and adoption of CMD management practices

**Fig. A1:**
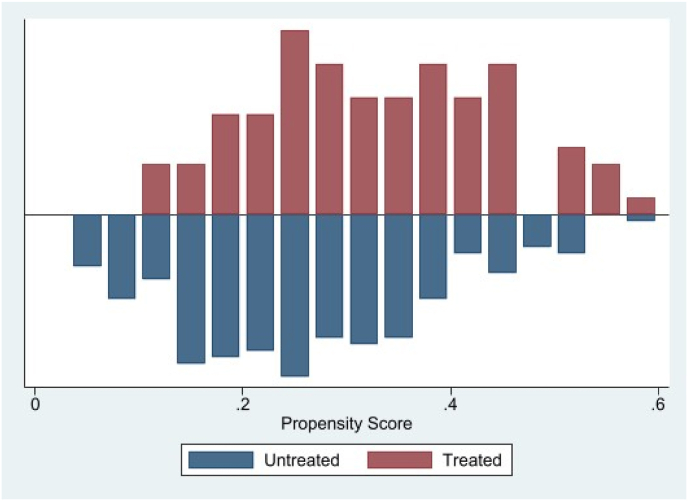
A graph illustrating substantial overlaps in the propensity score distribution between participants and non-participants in awareness campaigns

This section presents the results of Propensity Score Matching (PSM) regarding the effects of participation in awareness campaigns on the level of knowledge and adoption of CMD management practices, after correcting for selection bias due to observable differences between participants and non-participants. Prior to estimating the average treatment effect on the treated (ATT), a multicollinearity test was conducted on the variables included in the model. Highly correlated variables were excluded, retaining only those with no strong correlation. Subsequently, the presence of common support conditions and covariate balance was verified. Figure A1 in the appendix demonstrates substantial overlap in the propensity score distribution between participants and non-participants in the awareness campaigns. These conditions ensure that the groups are comparable, and causal effect estimates are reliable.

As previously stated, the ATT is a statistical measure that quantifies the average difference between the actual outcomes of farmers who participate in awareness campaigns and the outcomes they would have achieved in the absence of such campaigns. The results presented in Table 5 demonstrate a significant association between participation in awareness campaigns and improvements in CMD knowledge levels and the adoption of management practices. The ATT indicates that participation in the campaigns contributes to an increase of 0.13 and 0.17, respectively, for CMD knowledge level and the adoption of its management practices. In particular, the awareness campaigns enabled participating farmers to enhance their CMD knowledge level and the adoption of management strategies by 32.5 % and 36.17 %, respectively. The disaggregated results for CMD knowledge level are presented in Appendix Table A2.

**Table 5:** Matching estimates using nearest neighbour technique for the impacts of awareness campaign.

Variables	Mean of the variables		ATT	ATT (%)
	Participants	Non-Participants		
General knowledge related to CMD	0.53	0.40	0.13∗∗∗	32.5
General adoption of CMD management practices	0.64	0.47	0.17∗∗∗	36.17

∗∗∗p < 0.01, ∗∗p < 0.05, ∗p < 0.1.

### 3.4 Robustness checks

In order to assess the robustness of the ATT estimates, two alternative matching algorithms were employed: Radius Matching (RM) with a caliper of 0.05 and Kernel Matching (KM) with a default bandwidth of 0.06. Table 6 presents the impact estimates from these three matching algorithms for awareness campaigns. Overall, the comparison of effect estimates between the two alternative methods and the Nearest Neighbour Matching (NNM) approach is consistent. This substantial similarity in results suggests that our impact estimates remain robust regardless of the matching method used. Additionally, the pseudo R2 is low and equals 0.0620.

**Table 6:** Comparison of ATT estimates by algorithms.

Variables	Estimation algorithm		
	Nearest Neighbour Matching (NNM)	Radius Matching (RM)	Kernel Matching (KM)
General knowledge related to CMD	0.13	0.13	0.11
General adoption of CMD management practices	0.17	0.17	0.19

Given that PSM relies on the assumption of no bias related to observable characteristics, the results may be subject to bias if unobserved differences (hidden bias) exist between participants and non-participants in awareness campaigns, potentially influencing the outcome variables. To ascertain the sensitivity of the estimated ATT to this hidden bias, Rosenbaum bounds (critical gamma levels, Γ) were calculated. These bounds quantify the extent to which unobserved characteristics, which influence the decision to participate in awareness campaigns, would need to differ in order to impact ATT estimates. The results indicate that the significant effect of participation in awareness campaigns on CMD would only be questioned if the value of Γ reached 3.2. In other words, the estimated significant effects of awareness campaigns are quite robust to hidden bias.

## 4 Discussion

The objective of this study is to evaluate the impact of awareness campaigns conducted by WAVE on farmers’ knowledge of CMD, its incidence in the fields, and the adoption of management practices. The results indicate a significant improvement in farmers' knowledge, with a 32.5 % increase in their understanding of CMD and a 36.17 % increase in the adoption of disease management practices in the treated group. These findings are consistent with those observed in previous studies. For instance Ref. [46], demonstrated that in Rwanda, exposure of maize farmers to combined extension channels (plant health meetings, radio broadcasts, SMS messages) led to a better ability to identify Fall Armyworm (FAW) and a higher adoption of management practices. Similarly [47], observed in Bangladesh that training vegetable farmers in Integrated Pest Management (IPM) improved their knowledge and reduced the excessive use of pesticides, while promoting the adoption of good IPM practices. These examples highlight the importance of awareness campaigns in improving agricultural knowledge, correcting misconceptions, and encouraging the adoption of good practices.

However, despite progress, some aspects of CMD remain poorly understood. For example, the modes of transmission and causes of the disease are still not well grasped by farmers. This lack of understanding can be attributed to the technical nature of the awareness campaigns, which addressed complex scientific concepts that were difficult for all farmers to assimilate. Furthermore, the limited duration of the campaigns may not allow for a deeper exploration of these aspects or for addressing all farmers' questions. There are also local perceptions regarding the causes of CMD, which are sometimes attributed to factors such as soil degradation, insufficient rainfall, or the use of phytosanitary products.

Regarding the adoption of CMD management practices, while significant progress has been made, some essential practices remain poorly adopted, such as the use of healthy cuttings and the application of PlantVillage Nuru [48,49]. These practices have adoption rates of 40 % and 41 %, respectively, among participants, suggesting barriers to their adoption. The high cost of healthy cuttings presents a major obstacle for smallholder farmers, who are often financially constrained [50], while the limited availability of these cuttings in certain regions further complicates their adoption. Indeed, given the high incidence of the disease in cassava fields, it becomes challenging for farmers to obtain healthy cuttings in adequate quantities during the sorting stage. Additionally, the tradition of receiving seeds for free exacerbates this issue. As for the PlantVillage Nuru application, its low adoption may be related to its recent introduction in the region, farmers' lack of awareness of the tool, and the absence of effective information dissemination channels [51]. The lack of awareness and the limited diffusion of information through elite farmers hinder its large-scale use [48]. Furthermore, the unavailability of smartphones in rural areas and the low level of eduction among farmers make it difficult to the use this technology.

To maximize the effectiveness of awareness campaigns, it is recommended to adopt a decentralized approach by organizing training sessions at the local community level. These sessions should be tailored to the specific needs of farmers, taking into account their socio-economic realities and local challenges. Furthermore, the establishment of plant clinics, as advocated by Ref. [28], would provide immediate technical support. The combined use of mass media, such as community radio and SMS messages, particularly in rural areas, would further promote the dissemination of good agricultural practices [29,52].

Regarding the incidence of CMD, symptoms were observed in more than 60 % of the inspected cassava fields. Several factors explain this situation, notably the high pressure from whiteflies, the primary vector of the disease [17]. showed in Benin that the Couffo (PDA 5) and Mono (PDA 7) departments experienced high whitefly pressure, with 15.03 and 13 flies per plant, respectively. The limited availability of healthy planting material is another critical factor, leading farmers to use infected cuttings. Although awareness efforts have been made, the lack of adequate management of whiteflies and effective mechanisms for acquiring healthy cuttings complicate the fight against CMD. Therefore, it is crucial to implement strategies for managing whiteflies and establish distribution networks for virus-resistant varieties to enhance the effectiveness of awareness campaigns and reduce the prevalence of the disease.

## 5 Conclusion

This study was based on collected data from 305 cassava farmers distributed across three Agricultural Development Poles (PDAs) in Benin. The objective was to assess the impact of awareness campaigns on CMD. Three key aspects were assessed: CMD knowledge, adoption of disease management strategies, and field incidence of CMD.

To mitigate the potential selection bias, we employed the Propensity Score Matching (PSM) method to analyze these impacts. In our impact analysis approach, we followed standard PSM steps, including identifying the propensity score for each farmer based on identified covariates, determining common support, selecting matching algorithms, verifying match quality, estimating the average treatment effect, and conducting sensitivity analysis. This method allowed us to compare farmers who participated in awareness campaigns with a similar control group in terms of observable characteristics.

Overall, the results suggest that the awareness campaigns contributed to improved CMD knowledge among farmers, facilitated the adoption of disease management practices, and reduced the incidence of CMD in the fields. These results suggest that integrating awareness campaigns into agricultural policies could contribute to global efforts in combating cassava viral diseases, including CMD.

However, the results also reveal that CMD symptoms are present in the majority of surveyed fields. It is therefore imperative that existing awareness campaigns are enhanced. It is recommended that these campaigns be enhanced by organizing decentralized training at the community level, establishing plant clinics, using SMS messages at the beginning of the agricultural season, and dissemining audio recordings through NGOs, local radio stations, and public announcers. These measures will facilitate the dissemination of information to a larger number of farmers. In addition to these measures, it is crucial to consider biological factors such as the whitefly population and the scarcity of healthy plant material, which significantly undermine the effectiveness of awareness campaigns.

Finally, It is important to note that our study is not without limitations. Firstly, the cross-sectional survey data employed in this study does not permit an investigation of the long-term dynamics of participation in awareness campaigns or the incidence of CMD in farmers' fields. Secondly, the data used in this study was derived from a single country in which the awareness campaigns were conducted. Consequently, further research using longitudinal data and data from different geographical contexts would provide better insights into disease management implications. Such future studies could focus on the impact of awareness campaigns on CMD incidence and severity, as well as on farmers' yield and income.

## CRediT authorship contribution statement

**Dèwanou Kant David Ahoya:** Writing – review & editing, Writing – original draft, Software, Methodology, Formal analysis, Data curation, Conceptualization. **Jacob Afouda Yabi:** Writing – review & editing, Validation, Supervision, Methodology, Conceptualization. **Jerome Anani Houngue:** Writing – review & editing, Investigation. **Serge Sètondji Houédjissin:** Writing – review & editing, Investigation. **Martine Zandjanakou-Tachin:** Writing – review & editing, Supervision. **Ettien Antoine Adjei:** Writing – review & editing. **Eveline Marie Fulbert Windinmi Sawadogo-Compaore:** Writing – review & editing, Supervision. **Justin Simon Pita:** Supervision, Resources, Project administration. **Corneille Ahanhanzo:** Supervision, Resources, Project administration.

## Funding

The authors declare financial support was received for reasearch, authorship, and publication of this article. This study was funded by the European Union (EU) through the Biorisks project executed by the Regional Center of Excellence for Transboundary Plant Pathogens, Central and West African Virus Epidemiology (WAVE) and the Conseil Ouest et Centre Africain pour la Recherche et le Développement Agricoles (CORAF), grant number: CORAF N°: SC001_MC001_UE-WAVE CRIS n° FOOD 2019/411–531 and the 10.13039/100000865Bill and Melinda Gates Foundation and the United Kingdom Foreign, Commonwealth, and Development Office (10.13039/501100020171FCDO; INV-002969; grant no. OPP1212988) to the Central and West African Virus Epidemiology (WAVE) Program for root and tuber crops, Université Félix Houphouët-Boigny (UFHB) to Université Abomey-Calavi, Benin. Under the grant conditions of the Foundation, a Creative Commons Attribution 4.0 Generic License has already been assigned to the author-accepted manuscript version that might arise from this submission.

## Declaration of competing interest

The authors declare that they have no known competing financial interests or personal relationships that could have appeared to influence the work reported in this paper

## Figures and Tables

**Table A1:** Farmers interviewed in each commune

PDA	Departments	Communes	Number of sampled farmers		
			Participants	Non-Participants	Total
PDA 5 (Zou- Couffo)	Couffo	Aplahoué	2	11	13
		Djakotomey	2	9	11
		Dogbo	1	10	11
		Klouékanmey	2	8	10
	Zou	Agbangnizoun	0	6	6
		Covè	3	7	10
		Ouinhi	3	11	14
		Zagnanado	3	9	12
PDA 6 (Plateau)	Plateau	Adja-Ouèrè	3	10	13
		Ifangni	6	4	10
		Kétou	5	9	14
		Pobè	4	12	16
		Sakété	2	15	17
PDA 7 (Ouémé- Atlantique- Mono)	Atlantique	Abomey-Calavi	6	6	12
		Kpomassè	1	10	11
		Toffo	5	6	11
		Tori-Bossito	5	9	14
		Zè	3	8	11
	Ouémé	Adjohoun	4	7	11
		Akpro-Missrété	6	5	11
		Adjarra	4	5	9
		Avrankou	2	9	11
		Bonou	3	10	13
		Dangbo	2	7	9
	Mono	Comè	3	9	12
		Houéyogbé	5	8	13
**Total**			**85**	**220**	**305**

**Table A2:** Disaggregated results of the Average Treatment Effect on the level of CMD knowledge

Variables	Mean of the variables		ATT	ATT en %
	Participants	Non-Participants		
Identification of CMD symptoms	0.98	0.95	0.03	3.16
Knowledge of the name of CMD	0.41	0.29	0.12	41.38
Knowledge of the causes of CMD	0.31	0.16	0.15	93.75
Knowledge of the modes of propagation of CMD	0.38	0.23	0.15∗∗∗	65.22
Knowledge of CMD management practices	0.60	0.36	0.24∗∗∗	66.67
General knowledge related to CMD	0.53	0.40	0.13∗∗∗	32.5

## Data Availability

Data will be made available on request.

## Abbreviations

CMD: Cassava Mosaic Disease
WAVE: Central and West African Virus Epidemiology
PSM: Propensity Score Matching
PDA: Development Poles
